# Anti-IL-5 and anti-IL-5 receptor therapy significantly improves quality of life and FEV1 values in patients with severe asthma

**DOI:** 10.1186/s13223-025-00979-y

**Published:** 2025-08-13

**Authors:** Anaiza Odalis Villalobos Alfaro, Haydee Carolina Gutiérrez Vargas, Juan Manuel Díaz, Jonathan Alvarez Pinto, Diana Cristina García Cambero, Eduardo Hernandez Cuellar, Julio Augusto Palma Zapata, Alondra Esthefanía Llamas Domínguez, Juliana Palma Zapata, Silvia Denise Ponce-Campos

**Affiliations:** 1 Department of Medicine, Cuauhtémoc University, Aguascalientes, Mexico; 2 Institute of Security and Social Services for State Workers, Aguascalientes, México; 3 Department of Microbiology and Immunology, University of Western Ontario, London, ON Canada; 4 Institute of Security and Social Services for State Workers, Zapopan, México; 5 Mexican Social Security Institute, Nayarit, México; 6 Department of Morphology, Autonomous University of Aguascalientes, Aguascalientes, México; 7 Department of Medicine, Autonomous University of Aguascalientes, Aguascalientes, México

**Keywords:** Asthma, Anti-IL-5, Anti-IL5R, ACQ5

## Abstract

In recent years, the use of monoclonal antibodies directed against interleukin-5 (anti-IL-5) and its receptor alpha (anti-IL-5R) has proven to be an effective therapeutic option for patients with severe asthma by reducing the number of eosinophils, which may promote disease remission. This study aimed to evaluate clinical improvement and remission in patients with severe asthma treated with anti-IL-5 and anti-IL-5R antibodies over a period of 12 months. A cohort study was conducted with 49 patients diagnosed with severe eosinophilic asthma and who did not respond to conventional treatment. During follow-up, medical control was performed every 3 months using spirometry, eosinophil counts, quality of life scales, and disease control. The results revealed an improvement in FEV1 after 3 months of treatment, with statistical significance at 12 months in patients treated with anti-IL-5 and at 9 months in those treated with anti-IL-5R. In addition, better perceptions of asthma control and quality of life were observed, with significant differences at 6 and 12 months. Correlations between spirometry and ACT, ACQ, and AQLQ reflect a progressive recovery of well-being and function. Finally, the remission rate was 41.1% with anti-IL-5 treatment and 47.3% with anti-IL-5R treatment after one year of follow-up. These findings support the efficacy of anti-IL-5 and anti-IL-5R treatment in improving severe asthma control and patients’ quality of life, suggesting their key role in disease remission.

**Supplementary Information:**

The online version contains supplementary material available at 10.1186/s13223-025-00979-y.

## Introduction

Severe asthma is defined as asthma that remains uncontrolled despite optimized treatment with high doses of inhaled corticosteroids and long-acting beta-2 agonists (ICS-LABAs); asthma is a common disorder affecting approximately 7.8% of the U.S. population or 23 million Americans [1]. Approximately 10% of adults and 2.5% of children with asthma develop severe asthma, which negatively impacts their quality of life and increases the risk of airflow limitation, exacerbation, hospitalization, and even death [2]. Additionally, this pathology encompasses various clinical phenotypes varying by age of onset, presence of allergies, and coexisting conditions [1, 2]. These phenotypes include type 2 (T2) and non-T2 asthma, with significant differences in treatment response, particularly to inhaled corticosteroids (ICSs) [3]. T2 asthma primarily features eosinophilic airway inflammation in 50% of cases associated with increased blood eosinophil counts, whereas non-T2 asthma includes neutrophilic and pancigranulocytic asthma [1, 4–7].

On the other hand, difficult-to-treat asthma is defined as uncontrolled asthma despite adequate treatment. A number of factors, such as poor adherence to treatment or the misuse of inhalers, may lead to treatment failure and to an uncontrolled patient [3]. It is important to distinguish between severe asthma and difficult-to-control asthma, which is caused by modifiable factors such as poor inhalation technique, poor treatment adherence, or the presence of comorbidities such as chronic rhinosinusitis or obesity [2]. To differentiate between uncontrolled and controlled asthma, tools such as the Asthma Control Questionnaire (ACQ5) and Asthma Control Test (ACT) are used in adolescents and adults to assess asthma control and classify patients into different levels on the basis of the reported symptoms [8].

There are different treatments for severe asthma patients. Recently, monoclonal antibodies such as anti-interleukin-5 (Anti-IL-5) and anti-interleukin-5 receptor alpha (Anti-IL5R) have been used to control patients with severe asthma [2]. However, few studies have focused on patients treated with these therapies in real-life settings, especially regarding clinical remission and complete remission, which may involve discontinuation of the therapy. Notably, both antibodies reduce the number of eosinophils, while the anti-IL-5R antibody also depletes basophils [2].

This study aims to compare current epidemiological and follow-up data on biomarkers, respiratory function tests, quality of life scales, and annual remission in patients with severe eosinophilic asthma treated with anti-IL-5 or anti-IL-5R.

## Materials and methods

This cohort study was conducted at ISSSTE Aguascalientes General Hospital, Valentín Gómez Farías Zapopan Hospital, and the Mexican Institute of Social Security Tepic Hospital between February 2021 and February 2023. The study included patients aged 18 to 99 years with confirmed severe eosinophilic asthma type 2, regardless of sex, who did not respond to conventional treatment with high-dose corticosteroids. Those who did not meet the definition of severe asthma, experienced an exacerbation at baseline, were pregnant, or had conditions that could mask asthma control symptoms, such as ischemic heart disease, neurodegenerative diseases, or concomitant cardiorespiratory conditions, were excluded. The study was approved by the ethics committee (Approval No. 2024-RCEI-9), and all participants provided informed consent.

Patients who met the definition of severe eosinophilic asthma characterized by uncontrolled asthma despite adequate adherence to inhaled therapy, high-dose ICS/LABA, and controlled modifiable risk factors were included [3]. In addition, patients received at least one year of treatment with anti-IL-5 or anti-IL-5R monoclonal antibodies (benralizumab 30 mg every 4 weeks and then every 8 weeks or mepolizumab 100 mg every 4 weeks) at their respective hospitals. This therapy has been extensively evaluated in controlled clinical trials, establishing that the greatest therapeutic effect is observed with those doses [4–7, 9–27].

**Fig. 1:**
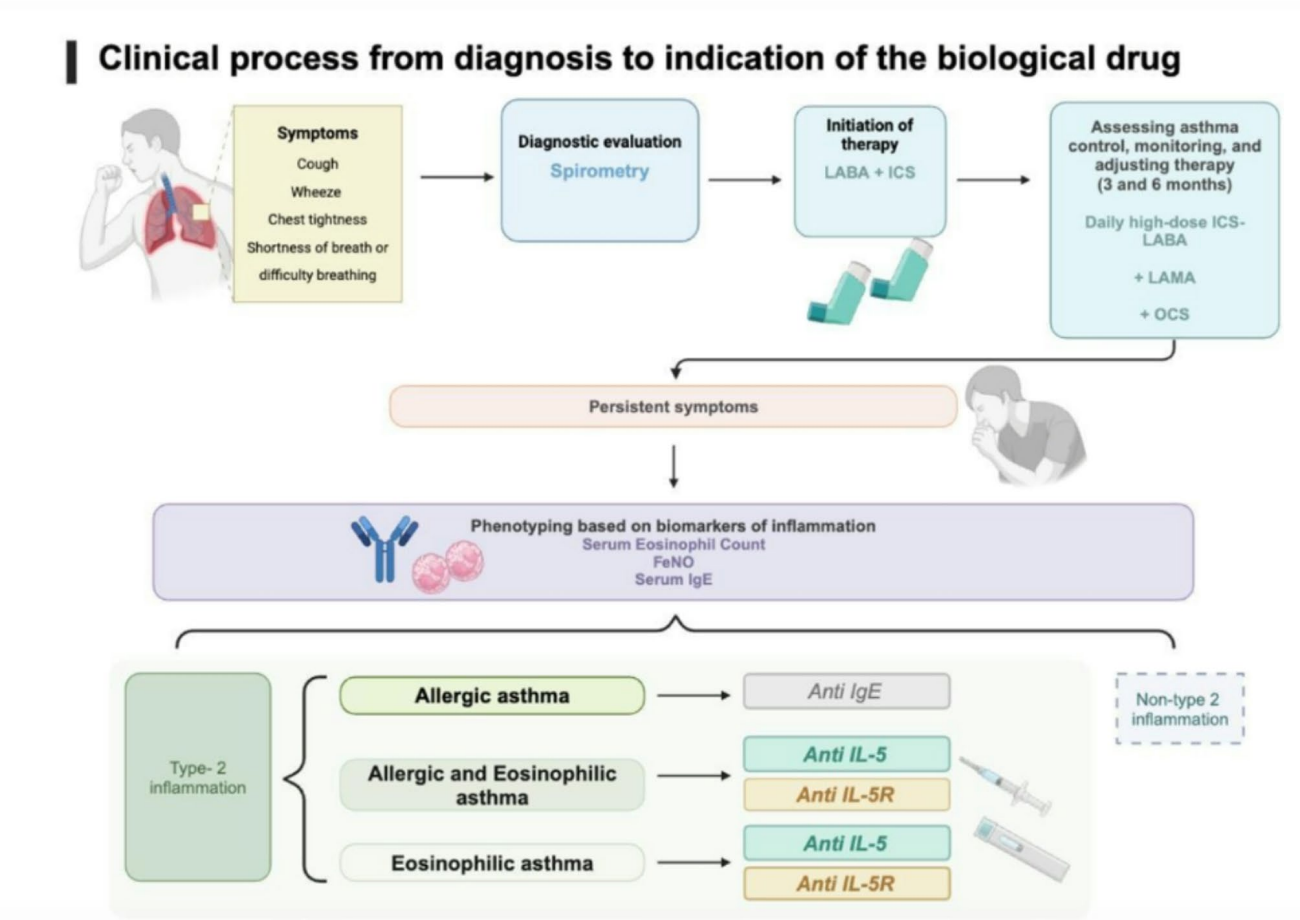
Clinical asthma patient flowchart used in this study from diagnosis to treatment

**Fig. 2:**
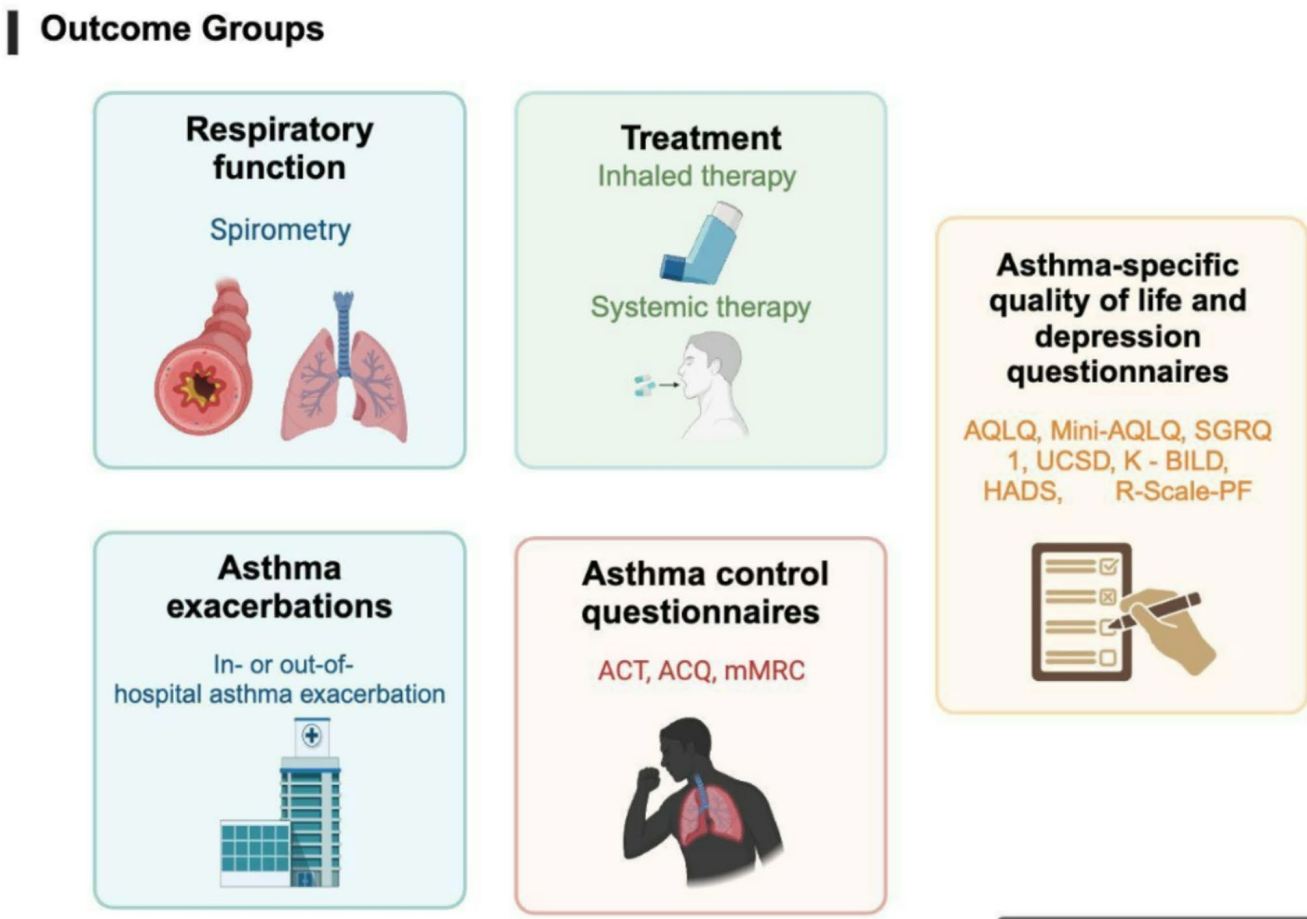
Asthma patients’ outcomes in consideration for this study

**Fig. 3:**
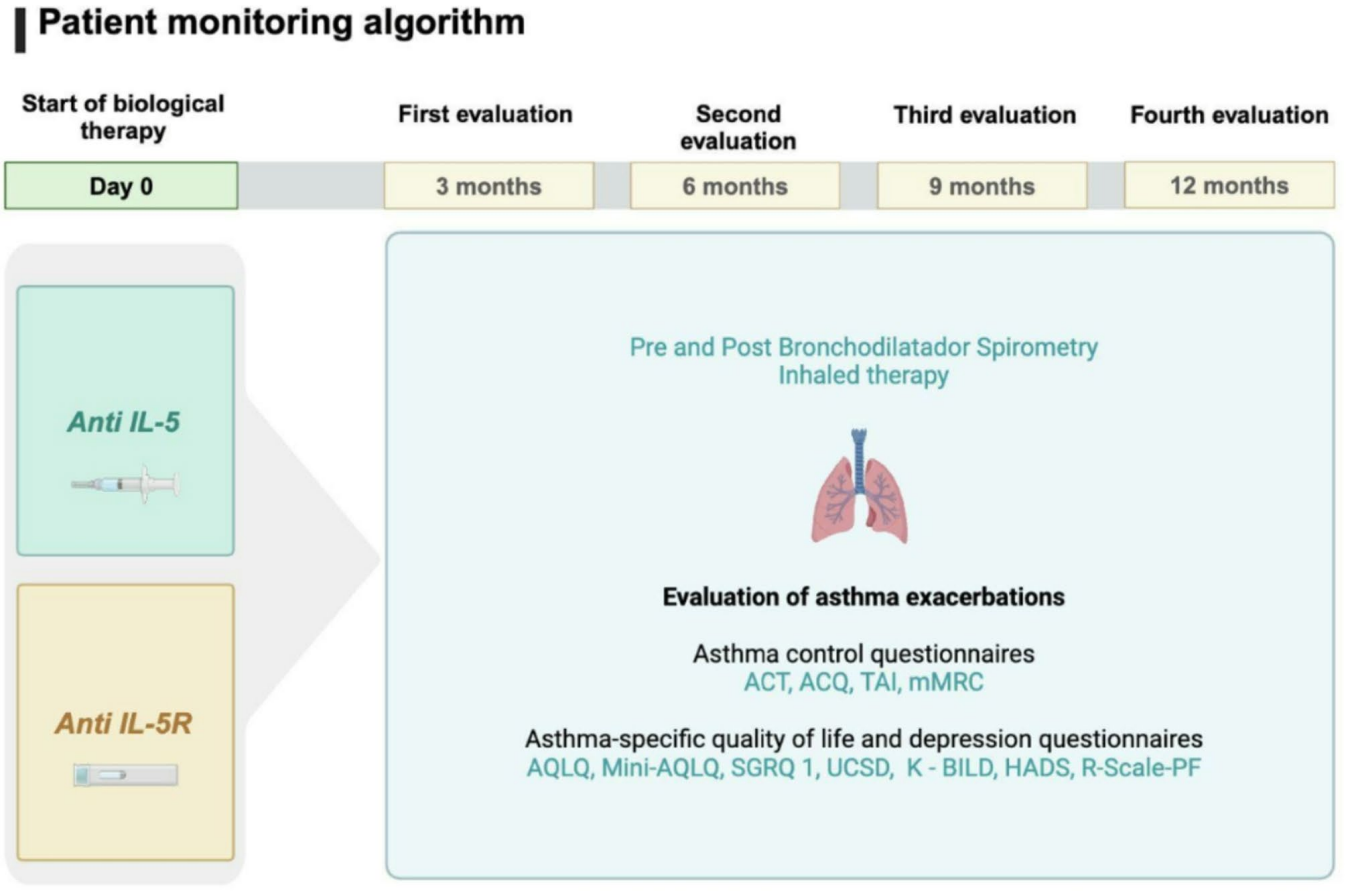
Algorithm used to monitor patients in this study for a period of 12 months

During the follow-up period, every three months, medical control, including spirometry tests and eosinophil counts, was performed for each patient. Quality of life (Table 1) and asthma control scales (Table 2) were applied at each visit to determine the patients’ quality of life, treatment adherence, and biological treatment. (Figures 1, 2 and 3)

**Table 1:** Quality-of-life tools used in this study

Test/tool	Characteristics	Scores/Interpretation
Asthma Quality of Life Questionnaire (AQLQ)	It is an instrument to assess the quality of life in disease through physical and emotional impact¨. It has 32 items corresponding to 4 dimensions of health: limitations of habitual activities (11 items), symptoms (12 items), emotional function (5 items), and environmental stimuli (4 items) [28]	0 = good health-related quality of life 10 = poor health-related quality of life
Mini Asthma Quality of Life Questionnaire (mini AQLQ)	It consists of 15 questions, grouped into 4 dimensions: symptoms (5 items), activity limitation (4 items), emotional function (3 items) and environmental stimuli (3 items) [29]	1 = Lower degree of disability 7 = greater degree of autonomy
Saint George’s Respiratory Questionnaire (SGRQ- 1)	It has 73 items and 3 domains: symptoms, activity, and impact of the disease on daily life. [30, 31]	0 = better health status 100 = worse health status
The University of California, San Diego Shortness of Breath Questionnaire (UCSD)	It is a self-administered questionnaire for dyspnea associated with activities of daily living (ADLs) featuring 24 items. Respondents are asked to rate themselves from 0 (“Not at all”) to 5 (“Maximum or unable to do so due to shortness of breath”) in two areas: 1. How short of breath they feel when performing various activities (21 items). 2. To what extent shortness of breath, fear of hurting oneself due to overexertion, and fear of shortness of breath limits them in their daily lives (3 items) [32]	Scores range from 0 to 120, with higher scores indicating greater dyspnea.
The King’s Brief Interstitial Lung Disease (K-BILD)	It is a questionnaire on health-related quality of life (HRQoL), a specific measure of interstitial lung disease (ILD), which comprises 15 items. It has three domains: psychological (KBILD-P), dyspnea and activities (KBILD-B), and chest symptoms (KBILD-C)) combined into a total score (KBILD-T) [28]	Score ranges from 0 to 100; 100 represents the best state of health.
Modified Dyspnea Scale from British Medical Research Council (mMRC)	It is used to establish a baseline in respiratory impairment due to dyspnea. It has 5 items (0: absence of dyspnea, 1: dyspnea when walking fast on the flat, 2: dyspnea that limits the pace of other people, 3: dyspnea that causes resting when walking approximately 100 m, 4: dyspnea that prevents leaving the house) [33]	With grades from 0 to 4, where the highest score expresses a greater functional limitation.
Hospital Anxiety and Depression Scale (HADS)		In each subscale, the score obtained is interpreted according to the following criteria: * 0–7 normal * 8–10 probable case * 11–21 case of anxiety or depression

**Table 2:** Asthma control tools used in this study

Test/tool	Characteristics	Scores/Interpretation
Asthma Control Test (ACT)	It assesses frequency of respiratory distress and general asthma symptoms, use of rescue medications, effect of asthma on daily functioning, and overall self-assessment of asthma control [34].	Scores range from 5 (asthma control) to 25 (complete asthma control). An ACT score > 19 indicates well-controlled asthma.
Asthma Control Questionnaire **(ACQ)**	A questionnaire that helps measuring the adequacy of asthma control that occurs spontaneously or because of treatment. The questionnaire consists of 5 questions [35].	Each question is scored from 0 to 6. The points are added up and divided by 5. According to the result: * Less than or equal to 0.75: Adequate asthma control. *0.75 to 1.50: Partially controlled asthma. * Over 1.50: Inadequate asthma control
Inhaler adherence test (TAI)	Questionnaire aimed at patients with asthma and COPD that allows us to identify patients with low adherence, establish the intensity of adherence (good, intermediate or poor), and provide guidance on the patient’s time or pattern of noncompliance. It is made up of 10 questions [36].	The score ranges from 1 (low compliance) to 5 (best compliance) The score provides a total ranging from 10 (minimum) to 50 (maximum)

Clinical remission was considered in patients with sustained absence of asthma symptoms (ACQ < 1 or ACT > 20), without exacerbations in the last year (they were assessed in a questionnaire, where they were intentionally asked if they had used systemic steroids or use of rescue drugs and were defined as yes or no) and lung function was optimized with post-bronchodilator FEV1 > 80%) [37]. This was evaluated only with spirometry with Easy One PC equipment, performed by a pulmonologist, previously calibrated equipment, accepting tests with grade A or quality B. Due to study limitations, pathologic complete remission was not assessed.

Statistical analysis. The data obtained in this study were analyzed, and the results were plotted via GraphPad Prism 8.0 software. Descriptive statistics were used to analyze demographic data and remission percentages obtained from patients. Clinical data from the spirometry-FEV1 (percentage as well as the Z-point score), ACT, and ACQ instruments were analyzed via paired RM one-way ANOVA. Quality of life was analyzed via the AQLQ, mini-AQLQ, SGRQ-1, UCSD, K-BILD, and HADSR tests and compared with the paired Friedman test and Dunn’s multiple comparisons. The difference between population data and *p* values was obtained and plotted in each figure.

## Results

After completing the 12-month schedule defined in this study, which included and compared data from patients who underwent all diagnostic tests, a population of 49 patients (100%) are shown in Table 3. It should be noted that it was established. In total, 73.4% of the patients were women and 26.5% men. Regarding age, the mean age of the population was 56.8 years (range: 42–74; SD: 14.7). A mean body mass index of 29.73 (range: 20–53; SD: 6.1). Nasal polyps were found in 18.4% of patients. Fourteen patients (28.6%) reported tobacco use, while 35 (71.4%) did not. In this study, 17 patients were treated with anti-IL-5 antibodies (35%) and 32 individuals with anti-IL-5R antibodies (65.3%). Quantification of baseline eosinophils prior to initiation of treatment in the anti-IL-5 group revealed a mean of 521.8/1 × 105 cells/µL (SD: 297.1), and for the anti-IL-5R group, the mean number of cells was 885.6.3/1 × 105 cells/µL (SD: 568.1). In this study, the following results belong to the two groups mentioned above.

**Table 3:** General characteristics of the study population

Variable		Mepolizumab	Benralizumab
Total of participants nº		17	32
Sex, male		Male: 2 (11.8%)	Male: 11 (34.4%)
Age		Mean: 58.1 years ± 15.8	Mean: 56.0 years ± 13.4
Body Mass Index (BMI)		Mean: 146.6 ± 131.5	Mean: 134.9 ± 127.9
Time Since Asthma Diagnosis (Months)		Mean: 181.8 ± 202.5	Mean: 173.2 ± 374.4
Nasal Polyps		3 (17.6%)	6 (18.8%)
Tobacco Use		Yes: 7 (41.2%)	Yes: 7 (21.9%)
Smoking Index		Mean: 5.3 ± 8.1	Mean: 3.8 ± 10.6
Pre-bronchodilator FEV1 (%)		Mean: 63.1 ± 16.8	Mean: 62.9 ± 14.3
Pre-bronchodilator FEV1 (Z-score)		Mean: -2.7 ± 1.5	Mean: -2.2 ± 1.2
Post-bronchodilator FEV1 (%)		65.6 ± 18.3	Mean: 66.4 ± 15.2
Post-bronchodilator FEV1 (Z-score)		Mean: -2.6 ± 1.5	Mean: -2.1 ± 1.2
GCS Use (yes)		12 (70.6%)	18 (56.3%)
Serum eosinophils (cells/mm³)		Mean: 521.8 ± 297.1	Mean: 885.6 ± 568.1
Serum IgE (UI/mL)		Mean: 1,784.4 ± 2,093.3	Mean: 2,710.0 ± 10,685.4
Exacerbations in the year before MAB		Mean: 1.7 ± 1.6	Mean: 2.5 ± 1.5
GCS	3 months	5 (29.4%)	8 (25%)
	6 months	3 (17.6%)	4 (12.5%)
	9 months	4 (23.5%)	4 (12.5%)
	12 months	7 (41.2%)	6 (18.8%)

The quantitative variables are expressed in mean and standard deviation, on the other hand, the qualitative variables in frequency and percentage

**Fig. 4:**
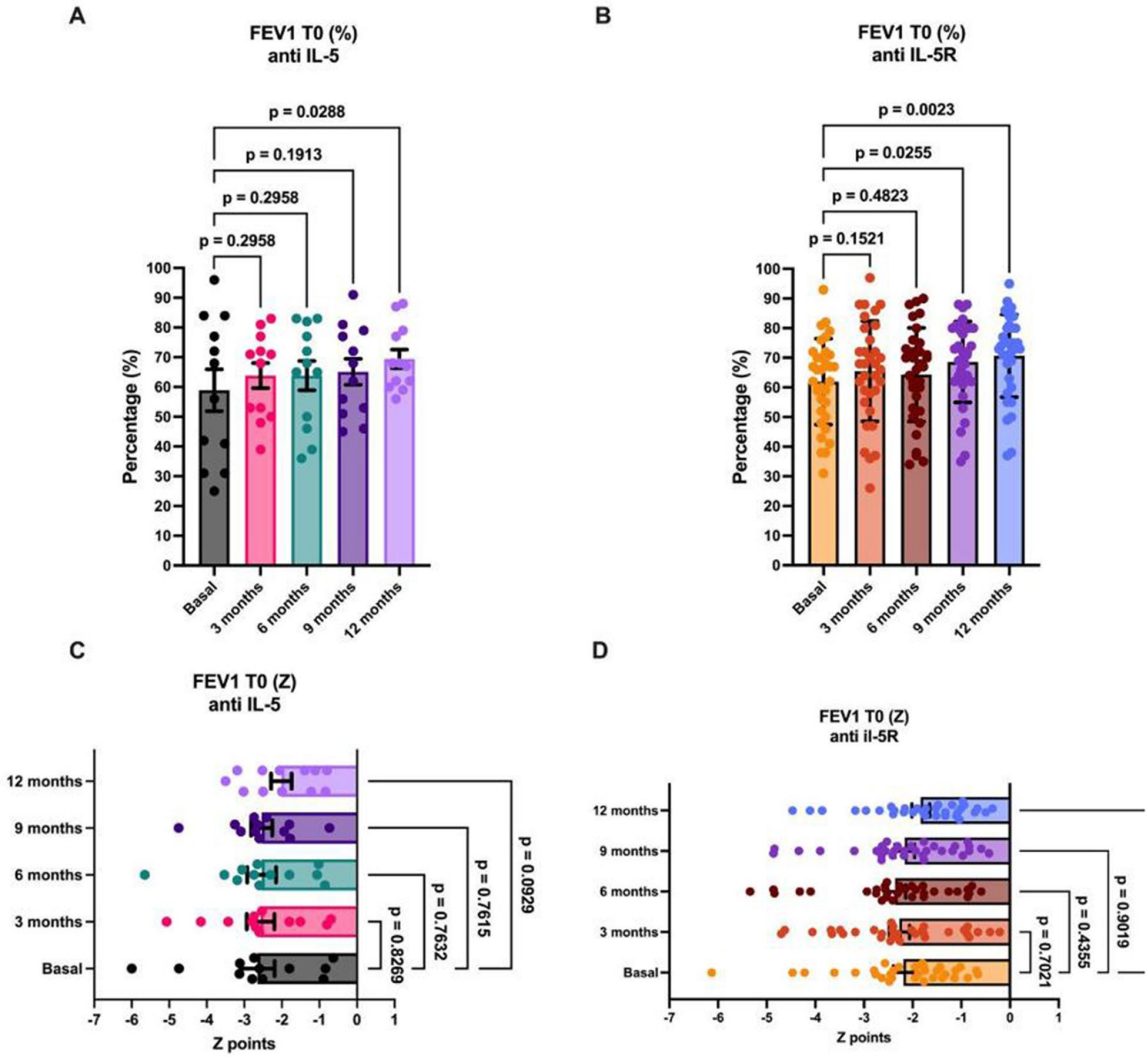
Lung capacity analysis after treatment of patients with anti-IL-5 and anti-IL-5R. Lung capacity was evaluated via the FEV1 with spirometry. (**A**) The percentage of lung capacity in the anti-IL-5 group improved beginning at 3 months, continued to increase until the final timepoint of the timeline, and was statistically significant at that point. In the anti-IL-5R group (**B**), this effect was also significant after 6 months of treatment. On the other hand, although the patients in both groups (**C** and **D**) presented a decrease in FEV1, neither the anti-IL-5 nor the anti-IL-5R group presented statistically significant differences in the Z score. Paired RM one-way ANOVA; the bars represent the geometric means, and each data point represents a single patient

Starting at the basal values and continuing the treatment throughout the year, the respiratory and lung capacity of the patients in each group was evaluated and compared. This study used the forced expiratory volume (FEV1) as well as calculated percentages and Z-points to measure lung function. First, anti-IL-5 therapy was analyzed (Fig. 4A). An improvement in the FEV1% was observed starting at 3 months of treatment; however, these changes were statistically significant after 1 year of treatment. The results corresponding to the anti-IL5R therapy (Fig. 4B) were similar to those of the previous treatment; however, these results were significant after 9 months in comparison with the basal values, and the improvement in pulmonary capacity continued at 12 months. On the other hand, the results obtained via the Z-point scale showed no change in these values, neither for the anti-IL-5 group (Fig. 4C) nor for the anti-IL5R group (Fig. 4D). Nevertheless, a reduction in the Z-point score was observed at the last timepoint in comparison with the baseline data. No significant difference was observed when comparing the results from each group in terms of the FEV1% and FEV1 Z-points (Supplementary Fig. 1). 

**Fig. 5:**
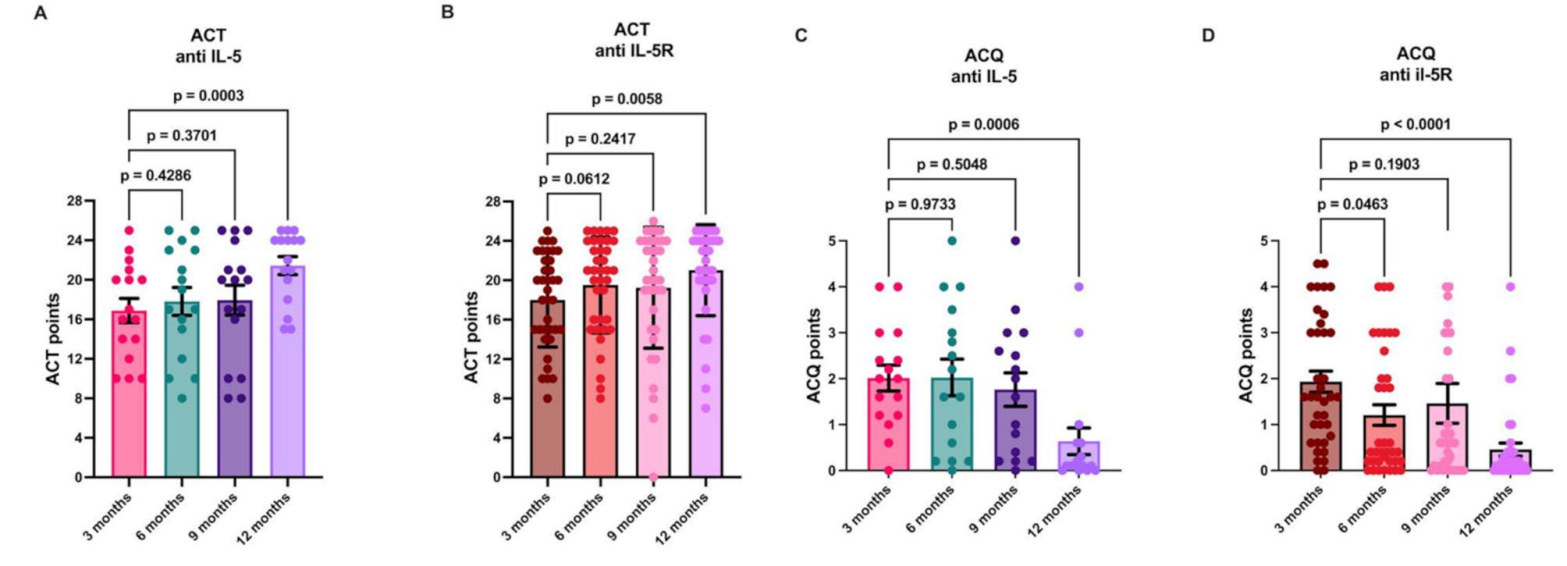
Control and improvement perceptions of asthma patients using ACT and ACQ tools. The patient´s perception of improvement using the ACT for the anti-IL-5 group (**A**) showed a significant improvement after 12 months of treatment. Similar results were observed for the anti-IL-5R group (**B**). Moreover, the ACQ results revealed significant improvement after 12 months for anti-IL-5 (**C**) and anti-IL-5R therapies (**D**). Paired RM one-way ANOV A, bars represent the geometric mean, while each data point represents a single patient

To determine the correlation between spirometry results and the perceived improvement of patients participating in this asthma protocol, we used the asthma control test (ACT) as well as the asthma control questionnaire (ACQ). The ACT results from the anti-IL-5 group showed a similar pattern to those of the spirometry group, demonstrating a significant time-dependent improvement at the end of 12 months in comparison to the first data point at 3 months (Fig. 5A). Asthma control perception was also enhanced over time in the anti-IL5R group (Fig. 5B). Furthermore, the ACQ results revealed a reduction in the scores for both groups (Fig. 5C and D), indicating significant differences only at 12 months. 

**Fig. 6:**
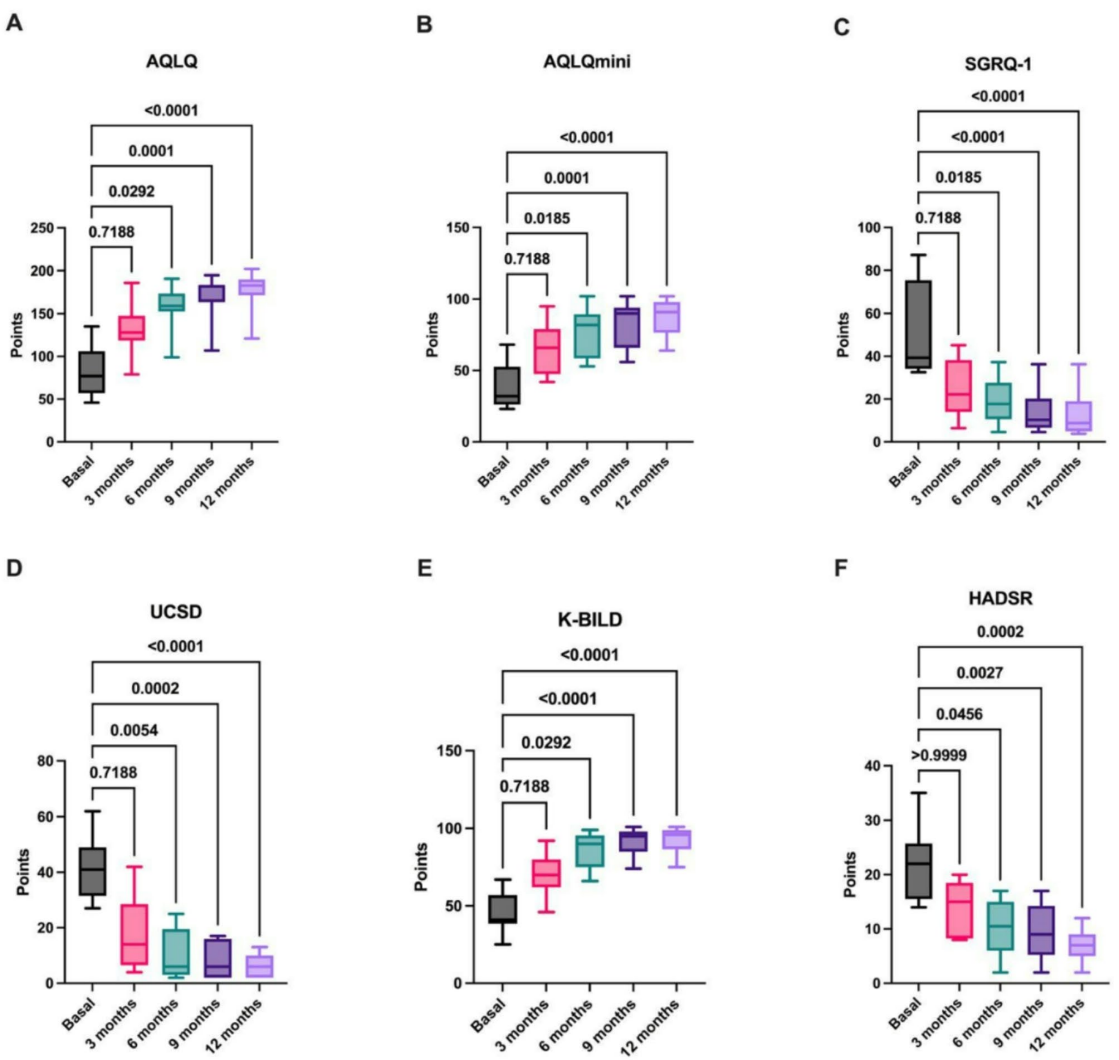
Assessment of quality of life in asthma patients treated with anti-IL-5 and anti-IL5R antibodies using AQLQ, mini-AQLQ, SGRQ-1, UCSD, K-BILD, and HADSR. To generate a global understanding of the quality-of-life progress by patients over time, this study collected data from six different instruments. There was statistical significance in all these tools after completing 6 months of treatment. Paired Friedman test, Dunn’s multiple comparisons

Next, to continue the evaluation of qualityoflife, repercussions after treatment with anti-IL-5 and anti-IL5R antibodies were similar to previously reported data. This study compiled the results of both groups for the six different questionnaires and compared them within the time points set every three months against the baseline data. First, the results from the asthma quality of life questionnaire (AQLQ) (Fig. 6A) revealed a significant difference at 6 months of treatment. Additionally, these results correlated with the results of the mini-AQLQ instrument used in this work (Fig. 6B). Afterward, the St. George’s Respiratory Questionnaire (SGRQ) was used to measure the overall wellness perceptions of the patients (Fig. 6C); interestingly, the patients mentioned an improvement in quality of life with treatment after 3 months. Similar results were observed for the UCSD, K-BILD, and HADSR questionnaires (Fig. 6D, E, and F), demonstrating a general improvement in different conditions that were reflected in the patients’ perceptions, as well as in their incorporation into normal life.

**Fig. 7:**
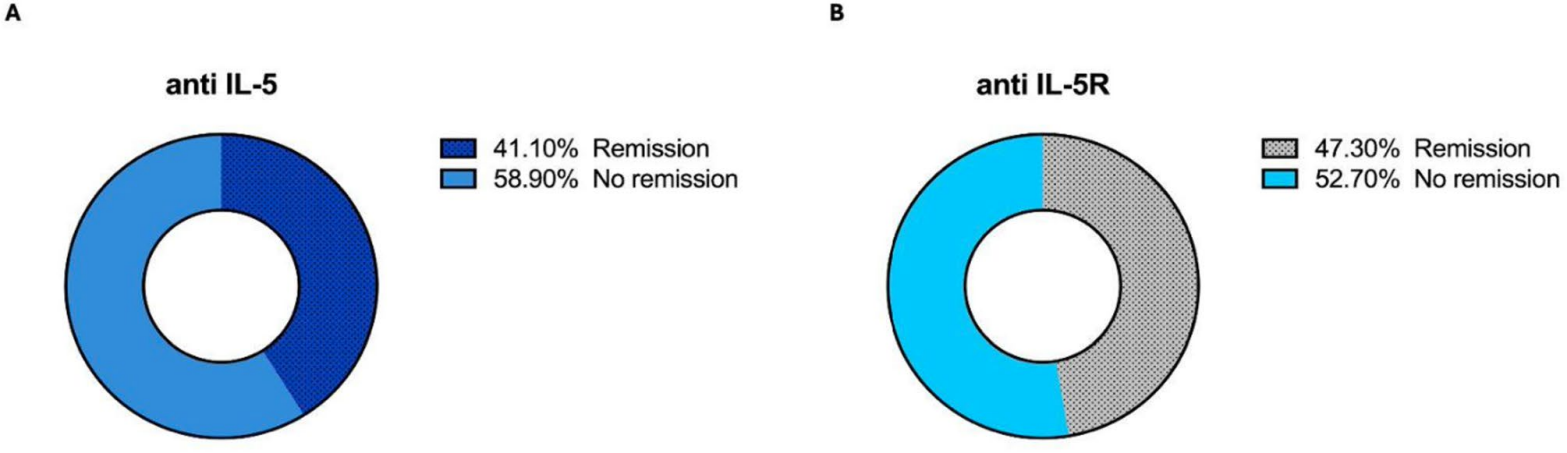
Remission percentage of patients treated after one year with anti-IL-5 or anti-IL5R antibodies. The percentage of patients who reported remission after completing 12 months of treatment with the anti-IL-5 drug (**A**) and with the anti-IL5R drug (**B**) was 41.10% and 47.30%, respectively. After comparing the data between both groups, no differences were observed

Finally, we evaluated the percentage of remission in the anti-IL5 and anti-IL5R treatment groups. According to the results, 41.10% of patients were in remission in the anti-IL-5 group (Fig. 7A). The other group, on the other hand, had a remission rate of 47.30% after a (Fig. 7B), of which none even had an indication for treatment with systemic glucocorticoid in the last year.

## Discussion

Severe asthma encompasses various phenotypes, including T2 and non-T2 asthma, with significant differences in treatment response, particularly to inhaled corticosteroids (ICS), or, in some cases, in the decision to initiate monoclonal pharmacologic therapy. T2 asthma is primarily characterized by eosinophilic airway inflammation in 50% of cases and is associated with increased blood eosinophil counts [2]. In contrast, non-T2 asthma includes neutrophilic and paucigranulocytic asthma. It is estimated that up to 83.8% of patients present an eosinophilic phenotype; however, a Latin American registry reported that 44% of patients had blood eosinophil counts above 300 cells/mm³ [38]. In this study, 45 patients were evaluated, 76% of whom were women, with ages similar to previously reported averages (55.7 years). Nasal polyposis was observed as a comorbidity in 22% of the patients, which is consistent with previously reported rates (9.8–35%). However, tobacco use was reported in 29% of cases in our cohort, a higher proportion than that observed in previous studies (XALOC 4%, PREPARE STUDY 9%, SIROCCO 1%) [7, 18, 19, 39]. This may have impacted pulmonary function, specifically FEV1.

In addition, this study reported that 65.3% of patients received anti-IL-5R therapy, which differs from other real-world studies in which most patients were treated with IL-5 inhibitors. However, our patients had high blood eosinophil counts, reaching up to 2,710 cells/mm³. This may have influenced the physician’s decision to initiate anti-IL-5R treatment with the goal of achieving a further reduction in eosinophilic burden. Previous studies have not demonstrated statistically significant improvements in FEV1 in patients treated with monoclonal therapies. In our study, a significant improvement in FEV1 was observed at 12 months in patients treated with anti-IL-5 and at 9 months in those treated with anti-IL-5R. These findings suggest that improvements in lung function may become evident in the long term. Further studies with longer follow-up periods are needed to assess the long-term efficacy of these therapies, particularly for patients receiving anti-IL-5.

Improvements in disease control, measured by ACT and ACQ-6 scores, were similar in both groups (anti-IL-5 and anti-IL-5R), consistent with findings from previous studies reporting significant improvements after 12 months of treatment compared to baseline [7, 9, 10, 14]. However, quality of life scores (AQLQ and mini-AQLQ) showed statistically significant improvements as early as 6 months after treatment initiation. The SGRQ-1 score decreased during the first 3 months, indicating clinically meaningful improvements in respiratory symptoms and overall perception of lung function. This positive effect persisted throughout follow-up, although no additional significant changes were observed beyond the early months, suggesting that the main benefits occur during the initial phases of treatment. This is consistent with other studies showing significant correlations between changes in SGRQ scores and other comparative measures, supporting its validity as a reliable and sensitive instrument for assessing health impairment in chronic airflow limitation diseases [40].

The UCSD questionnaire showed a similar trend, with significant improvements in scores from baseline to 3 and 6 months. Although the scores continued to improve, they did not reach statistical significance, possibly indicating stabilization of the intervention’s impact on daily functioning after the first few months. There is evidence supporting the use of this tool in interstitial lung disease, where it shows excellent agreement and moderate positive correlation with the MRC scale [41]; however, evidence is limited in severe asthma populations. K-BILD results showed statistically significant and sustained improvements across all follow-up points, highlighting the ongoing benefit of the intervention on overall well-being and reinforcing its importance in comprehensive disease management. Regarding the HADSR questionnaire, improvements were more modest compared to the other tools. Although significant score reductions were observed at 3 and 6 months, these changes were not sustained at 12 months. This suggests that while the intervention initially contributes to mental health symptom improvement, additional strategies—such as multidisciplinary evaluation by psychology or psychiatry—may be required to maintain those effects.

Early improvements in quality of life, despite delayed disease control, could be attributed to patients being highly symptomatic at baseline or receiving a late diagnosis of severe asthma. Therefore, even minimal improvements in disease control may have a significant and early impact on quality of life, potentially contributing to a higher rate of clinical remission.

In our study, clinical remission was achieved in 41.10% of patients treated with anti-IL-5 and in 47.3% of those treated with anti-IL-5R. This percentage is higher than previously reported, where the maximum remission rate reached 43.2% (REMI-M) [42, 43]. Another multicenter observational study reported complete remission in 30.12% of patients treated with anti-IL-5 and 40% of those treated with anti-IL-5R after 12 months of treatment [44]. A trend toward higher remission rates with anti-IL-5R therapy has been previously reported [7, 18, 19, 39], although the difference was not statistically significant. The high remission rate in our study may be related to better baseline lung function, fewer exacerbations, and a perception of uncontrolled disease at treatment initiation.

Quality of life improvements preceded clinical control in both groups, and earlier improvements were observed in the anti-IL-5R group. Long-term comparative studies are needed to establish statistically significant differences between these two treatments, as the observational nature of this study prevents establishing externally valid associations like those demonstrated in randomized controlled trials.

## Limitations of the study

This study has several methodological limitations that must be considered when interpreting the results. First, the small sample size, with a total of 45 patients, limits the generalizability of the findings, especially considering the clinical heterogeneity of patients with severe eosinophilic asthma. Second, the uncontrolled observational design, typical of a real-world cohort study, lacks placebo or comparative control group, which precludes definitive causal relationships between the biological intervention and the observed outcomes, as would be possible in a randomized clinical trial. Finally, an additional limitation was the absence of a standardized methodology to assess the progressive decrease of systemic glucocorticoids at defined doses and timeframes, since only one categorical variable indicating the presence or absence of such therapy was recorded, limiting the analysis of effective steroid reduction throughout treatment. however, it is observed that patients on mepolizumab initially used more systemic steroid than patients on benralizumab. This may explain why at 12 months they still had systemic steroids, although no dose reduction was evaluated. The decrease in the percentage of basal systemic steroids-12 months is 28.8% and benralizumab 37.5%, so in both the systemic steroid decreases.

## Conclusions

This real-world cohort study demonstrated that biological therapy with anti-IL-5 and anti-IL-5R monoclonal antibodies is effective in improving clinical asthma control, quality of life, and lung function in patients with severe eosinophilic asthma who do not respond to conventional treatment. Both therapies showed significant improvements in ACT, ACQ, and AQLQ scores, with over 40% clinical remission after 12 months of follow-up. Benefits were observed from the early stages of treatment—earlier with anti-IL-5R—and were sustained over time. These findings support the use of targeted therapies as a key tool to modify the clinical course of severe asthma, particularly in settings where optimizing outcomes and resources is essential. Further studies are needed to evaluate the long-term sustainability of these effects and their impact on complete disease remission.

## Electronic Supplementary Material

Below is the link to the electronic supplementary material.

Supplementary Material 1

## Data Availability

No datasets were generated or analysed during the current study.

## The following abbreviations are used in this manuscript

Anti-IL-5: Anti-interleukin-5
Anti-IL-5R: Anti-interleukin-5 receptor alpha
FEV1: Forced expiratory volume
ACT: Asthma Control Test
TAI: Inhaler adherence test
ACQ: Asthma Control Questionnaire
AQLQ: Asthma Quality of Life Questionnaire
mini-AQLQ: Mini Asthma Quality of Life Questionnaire
SGRQ: Saint George's Respiratory Questionnaire
UCSD: The University of California, San Diego Shortness of Breath Questionnaire
K-BILD: The King’s Brief Interstitial Lung Disease
HADS: Hospital Anxiety and Depression Scale
mMRC: Modified Dyspnea Scale from British Medical Research Council
IL-4: Interleukin-4
IgE: Immunoglobulin E
ICS: Inhaled corticosteroids
LABA: Long-acting beta-2 agonists
ANOVA: Analysis of variance
SD: Standard deviation
REMI-M: Maximum percentage of remission
