# Supplementary figures and images for: Anti-IL-5 and anti-IL-5 receptor therapy significantly improves quality of life and FEV1 values in patients with severe asthma
Source: Allergy Asthma Clin Immunol. 2025 Aug 13;21:34. doi: 10.1186/s13223-025-00979-y (PMC12351985; doi:10.1186/s13223-025-00979-y)

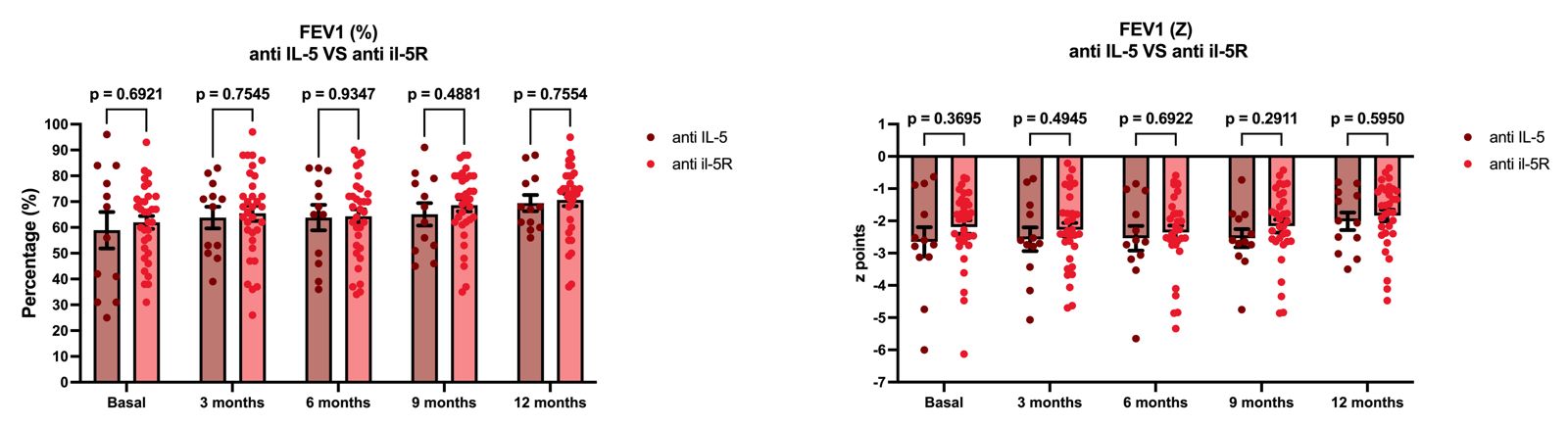

Supplement: Supplementary file 1 — Supplementary Material 1 [file 13223_2025_979_MOESM1_ESM.jpeg]
